# What determines the vote-seeking behavior of legislators in South Korea?

**DOI:** 10.1371/journal.pone.0304383

**Published:** 2024-07-10

**Authors:** Hanna Kim, Shang E. Ha

**Affiliations:** 1 Institute of Korean Political Studies, Seoul National University, Seoul, Republic of Korea; 2 Department of Political Science, Sogang University, Seoul, Republic of Korea; University of Catania, ITALY

## Abstract

Pre-existing studies suggest that legislators in small districts tend to be more responsive to the electorate than those in large districts, as accountability is typically clearer in a smaller setting. However, it is not clear whether the relationship between district size and legislators’ constituency-oriented spending holds in other electoral settings (e.g., South Korea), where pork barrel benefits tend to be determined at the administrative unit, not the electoral district. The present study hypothesizes that as the number of legislators in an administrative unit increases, they are less likely to try to appeal to the voters. Additionally, this study examines the moderating effects of partisan homogeneity and legislators’ seniority. The results from the statistical analysis of data from Korean National Assembly members confirm our hypothesis: the number of legislators in an administrative unit turns out to be negatively associated with their vote-seeking behavior. Such a relationship tends to be strong in administrative units co-represented by multiple parties and weak among newly elected legislators.

## 1. Introduction

Over the past few decades, a large amount of literature has examined the relationship between the electoral system and legislators’ personal vote-seeking behaviors [1–7]. An influential theoretical framework offered by Carey and Shugart [4] suggests that district size—measured by the number of representatives in a district—is negatively associated with the individual vote-seeking efforts of legislators. In theory, legislators are expected to behave in accordance with the interests of their constituents. But when multiple elected officials represent a single geographic area, they may care less about voters. This is because a higher number of representatives from the same constituency complicates recognition of individual efforts and hinders legislators from claiming credits for constituent-related services [4, 8]. Therefore, only in cases where the electoral district is small enough for the responsiveness of legislators to be clearly defined do they have strong incentives to cater to the electorate by bringing pork-barrel projects in [9–11].

Empirical evidence for Carey and Shugart’s theory abounds [5, 7, 12, 13], with some studies offering dissenting opinions [1, 6, 14–16]. Pre-existing literature seems to be well-established, but it generally fails to take diverse political systems into consideration. In some countries, for example, material benefits via pork-barrel projects (i.e., intergovernmental transfers from the central government to local ones) are typically allocated at the level of the administrative unit, not the electoral district. An administrative unit may be identical to an electoral district, but it is possible for an administrative unit to include multiple electoral districts. One cannot rule out the possibility that the negative relationship between district size and the individual vote-seeking behavior of legislators may not hold in some political systems.

For instance, in South Korea, densely populated cities are divided into multiple electoral districts, whereas rural areas with fewer people contain a relatively small number of districts. Both Chungju-si in Chungbuk and Suwon-si in Gyeonggi are a single administrative unit, respectively, but the former comprises one electoral district, while the latter is divided into five electoral districts. Legislators may have to pay attention not only to the size of the district but also to other factors (e.g., how many districts are included in an administrative unit and how politically diverse the administrative unit is) in their personal vote-seeking behaviors.

That said, a pair of nuanced hypotheses can be offered. In an area where the administrative unit aligns with a single electoral district, legislators are more likely to keep an eye on the needs of their constituents because it is clear who holds responsibility for local issues. Conversely, in an area where multiple electoral districts exist in one single administrative unit, legislators are likely to be less attentive to constituents’ needs because it is unclear who can claim credit for constituency services. The fundamental logic of these hypotheses is similar to the theoretical reasoning of previous studies on the relationship between district size and legislators’ individual vote-seeking endeavors.

In sum, a legislator whose district is one of the many districts in an administrative unit is less likely to work hard for his or her constituents than another legislator whose district is identical to the administrative unit. Two conditional factors are considered here. First, partisan homogeneity can serve as a moderator. An administrative unit composed of many electoral districts may be represented by legislators from one single party or legislators from multiple parties. In the example shown above, five elected officials in Suwon-si may belong to one single party or may be affiliated with two or more parties. Since the quality of constituency services affects the fate of incumbents (either individual legislators or political parties) in the next election, a legislator who co-represents an administrative unit with the same party elected officials is more likely to try to meet the expectations of voters than another legislator who co-represents an administrative unit with the out-party-elected officials.

Second, the seniority of legislators can serve as another moderating variable. Senior legislators may rely less on constituency-oriented services than junior legislators, primarily because seniority is a function of visibility, reputation, leadership, and legislative influence. A large and deep personal network of senior elected officials in their districts allows them to be free from pork barrel projects. Having said that, a senior legislator who co-represents an administrative unit with other elected officials is less likely to try to meet the expectations of voters than a junior legislator in the same setting.

The present study utilizes data from the Korean National Assembly members from 2012 to 2022. Results from statistical analysis support the above-mentioned hypotheses. The findings contribute to our understanding of how the negative relationship between the number of elected politicians within a geopolitical area and their personal efforts to appeal to constituents can be applied to a broader, more diverse political setting. Moreover, the findings regarding moderators add value to the existing literature, allowing for a more nuanced interpretation of the roles of partisan homogeneity in an administrative unit and the seniority of legislators.

## 2. Literature review

In order to explain legislators’ personal vote-seeking behavior, previous studies have paid attention to the size of the electoral district (e.g., [4]). As the size of the district increases (i.e., the number of legislators per constituency increases), legislators are expected to spend less to cater to the interests of their constituents. This is primarily attributed to the elevated costs and decreased efficacy associated with vote-seeking efforts in larger districts. In fact, in large districts, the dispersion of credits for allocated goods or services among multiple legislators is prevalent. Conversely, small districts facilitate more direct attribution of legislative responsibility to specific legislators or parties, particularly concerning initiatives such as pork provision. This clarity arises from the reduced number of legislators sharing credit, enabling voters to more readily identify and evaluate individual legislators’ efforts on behalf of constituents [8, 17].

From the perspective of public choice theory, this dynamic may align with “the law of 1/n” and a common pool problem, which traditionally link the number of decision-makers to project size and overall spending [10, 18]. In the realm of pork-barrel politics, the law of 1/n is interpreted as follows: as the number of districts (n) increases, each district absorbs a smaller fraction of a project’s costs (1/n) since national projects are funded from a communal pool. Consequently, each district tends to seek a larger share of the project. The law of 1/n implies that an increase in the number of districts (and thus legislators) can potentially result in escalated government expenditure as legislators aim to cater to their constituents and ensure reelection.

However, it is crucial to note that in public choice discourse, the “law of 1/n” is often cited in scenarios where individual legislators strive to attain advantages, such as pork-barrel projects, exclusively for their respective districts. This occurs within a single-member district framework where the clarity of the legislators’ responsibility toward constituents is evident, as each electoral district is represented by a distinct legislator. Therefore, the mechanism behind the increase in government spending may be attributed to the heightened clarity of responsibility as the number of constituencies, each represented by a single legislator, grows.

When multiple legislators represent the same electoral district, directly applying this principle becomes more complex. In such scenarios, the assumption that the number of legislators (represented as n) seeks to obtain 1/n (or more than 1/n) stakes does not perfectly align. With multiple legislators jointly representing the same constituency, each is inclined to pursue individual strategies and priorities as the responsibility for their constituents becomes decentralized [8, 17]. As a result, the dynamics of cooperation or competition among them may deviate from the assumptions of the law of 1/n. In sum, common pool problems are more likely to manifest when the overall electoral district magnitude is small so that the individual responsibilities of legislators are clearly defined [9, 10, 19].

In examining the relationship between the number of legislators in a geographic area and their personal vote-seeking efforts, we propose shifting focus from electoral districts to administrative units. This transition allows us to integrate the theory of legislators’ personal vote-seeking behavior with the perspective from public choice literature on the common pool problem. By considering scenarios where multiple legislators from each electoral district form a single administrative unit, we can gain a more comprehensive understanding of these dynamics. It is crucial to note that the distribution of intergovernmental transfers from central governments to local administrations operates based on administrative units rather than electoral districts [20, 21]. In essence, governmental resources are allocated through pork-barrel projects based on administrative boundaries, which can be likened to “a common pool,” rather than electoral constituencies.

A legislative district often intersects with other administrative units, and instances occur where multiple legislators from each electoral district represent a single administrative unit, especially if it is densely populated, ensuring demographic representation. As a result, when an administrative region is divided into multiple electoral districts, instances occur where any financial benefit, such as a grant from the central government, is divided among the multiple constituencies, with each receiving 1/n of the total. In such scenarios, legislators encounter their fellow legislators. This context resembles the dynamics observed in multi-member districts discussed in previous literature regarding district size and legislators’ constituency-oriented efforts [2, 8, 14, 17, 19]. We can leverage these situations to investigate whether and how legislators’ efforts in personal vote-seeking behavior are influenced by the presence of other legislators within the same geographic unit.

## 3. Theory and hypotheses

### 3.1. The number of legislators per administrative unit

Expanding existing literature exploring legislators’ personal vote-seeking behavior [4, 8, 17], we argue that as the number of legislators per administrative unit increases, the incentive for legislators to engage in personal vote-seeking endeavors may decrease. This is due to the reduced clarity in attributing credit for allocated resources or efforts to local politicians amid a higher number of legislators [8, 17].

Research in cognitive psychology has consistently shown that humans have cognitive limitations, particularly when it comes to distinguishing among numerous options [11, 22]. This implies a constraint in voters’ cognitive capacity, potentially leading to difficulties in accurately discerning which politicians should be credited for progress in their surroundings, especially when multiple legislators represent the same administrative unit. Consequently, in such contexts, legislators may exhibit reduced motivation to prioritize local issues, potentially leading to instances of free-riding on the efforts of other legislators aimed at advancing the region.

This argument is consistent with the fundamental logic found in the literature, which suggests that an increase in the number of actors corresponds to a decrease in their individual efforts [8, 17]. Moreover, it aligns with the perspective of public choice theory, which emphasizes legislators’ motivation to appeal to their constituents, particularly evident in smaller district sizes [10, 18]. Based on the assumption, we formulate the following hypotheses:

#### Hypothesis 1

An increase in the number of legislators within an administrative unit correlates with a decrease in the likelihood that legislators seek to appeal to voters in their district.

### 3.2. Partisan homogeneity as a moderator

Previous research has extensively explored the impact of district magnitude on legislators’ vote-seeking behavior within their constituencies [1, 4–7, 12–16]. However, there has been a notable lack of emphasis on differences in party affiliation among legislators. We suggest that the relationship between the number of legislators within an administrative unit and their personal vote-seeking efforts in their district may vary depending on the diversity of party affiliations among the legislators within that administrative unit. As the number of political parties increases within an administrative unit, resulting in lower partisan homogeneity, the clarity of representatives’ responsibility to address voters’ interests and demands becomes obscured. This stands in contrast to a scenario where a single administrative unit is represented by multiple legislators, all belonging to the same party.

A legislative district typically overlaps with other administrative units, and multiple legislators may represent a single, populous administrative unit. Within an administrative unit, legislators can recognize the presence of fellow representatives and discern their party affiliations. Since material resources, such as those allocated through pork-barrel projects, are distributed at the administrative unit level rather than the electoral district level, legislators engage in strategic thinking regarding credit-claiming competition. When a legislator shares party affiliation with other elected officials in an administrative unit, they can more easily collaborate and are more motivated to address constituents’ needs. This is because their responsibility as a party becomes more apparent, and dissatisfaction from voters regarding the outcomes of constituency services attributed to a legislator can collectively damage the reputation of the party as a whole. Consequently, the degree of partisan homogeneity within an administrative unit may moderate the correlation between the number of legislators per administrative unit and legislators’ constituency-targeted behaviors. Building upon this assumption, the following hypothesis can be suggested:

#### Hypothesis 2

The negative relationship between the number of legislators within an administrative unit and legislators’ personal vote-seeking efforts in their district is stronger when the administrative unit is represented by legislators from different parties than when it is represented by multiple legislators belonging to the same party.

### 3.3. Legislator’s seniority as a moderator

Moreover, previous literature has largely overlooked differences in seniority among legislators when exploring the relationship between district magnitude and legislators’ personal vote-seeking efforts. Similarly, studies grounded in public choice theory have not extensively considered legislators’ seniority when examining the number of legislators and their activities related to pork-barrel projects.

We propose that the negative relationship between the number of legislators within an administrative unit and legislators’ personal vote-seeking efforts in their district may be more pronounced among legislators with greater seniority compared to newly-elected counterparts. This is because senior legislators have had more time to cultivate relationships with constituents compared to legislators in their first term. Their established networks with local voters may offer a stronger political support base, potentially reducing their reliance on constituency-oriented services. Additionally, senior legislators often hold leadership roles or key committee assignments. Their longer tenure in office grants them greater visibility among constituents, legislative influence, and resource accessibility. This may diminish the need for extensive personal vote-seeking endeavors aimed at local voters. Therefore,

#### Hypothesis 3

The negative relationship between the number of legislators within an administrative unit and legislators’ personal vote-seeking efforts in their district is stronger among senior legislators than junior legislators.

## 4. Data and measures

### 4.1. Case selection and institutional backgrounds

This study selected South Korea as its case study for two main reasons. First, South Korea offers access to official accounting reports containing detailed data on legislators’ spending. Under the Political Funds Act, each legislator is mandated to submit these reports to the National Election Commission (NEC), disclosing property holdings, revenues, expenditures of political funds, and total spending in their district. This information is publicly available.

While previous studies on legislators’ personal vote-seeking efforts often rely on proxies such as time spent in the district or surveys gauging subjective views on resource allocation [2, 14, 17], there remains a notable lack of research utilizing legislators’ spending data, which enables a more objective assessment. Although recent exceptional work exists utilizing a dataset of legislators’ political spending, this research focuses on the relationship between legislators’ spending and their legislative behavior [23]. Surprisingly, few studies explore the connection between the number of legislators in an administrative unit and their spending, particularly focusing on constituency-oriented spending.

Second, to scrutinize the relationship between the number of legislators within each administrative unit and their constituency-focused spending and to validate the applicability of the law of 1/n, it is important to select a case employing the single-member district (SMD) system, where each legislator exclusively represents an electoral district. Although South Korea transitioned from the mixed member majoritarian (MMM) system to the mixed member proportional (MMP) system since the 21st National Assembly, it is crucial to note that South Korea has never adopted a dual candidacy rule, which permits candidates to run as direct candidates for their constituency and as party list candidates simultaneously. This stands in contrast to Germany’s use of MMP, where such a rule exists [9]. Therefore, despite being categorized as a mixed-member system, South Korea’s single-member district members (SMDs) operate under conditions similar to those of a pure SMD system. This institutional characteristic aligns with the objectives of the study.

In Korea, the administrative units (called *Si*, *Gun*, and *Gu*) consist of 255 units, while there are 253 single-member electoral districts (see Table 1). The geographic boundaries do not necessarily align with each other. Consequently, more than one legislator can represent a single administrative unit. It is also possible for a single legislator to represent multiple administrative units.

**Table 1:** Administrative units and electoral districts in South Korea.

Regions	Administrative Units (*Si*, *Gun*, *Gu*)	Electoral Districts	Population
Seoul	25	49	9,770,638
Busan	15	18	3,436,230
Daegu	7	12	2,458,138
Incheon	8	13	2,956,063
Gwangju	5	8	1,459,208
Daejeon	5	7	1,487,605
Ulsan	4	6	1,153,735
Sejong	1	2	320,326
Gyeonggi	48	59	13,104,696
Gangwon	18	8	1,540,445
Chungbuk	15	8	1,598,868
Chungnam	17	11	2,125,372
Jeonbuk	16	10	1,832,227
Jeonnam	22	10	1,875,862
Gyeongbuk	24	13	2,671,587
Gyeongnam	23	16	3,371,016
Jeju	2	3	667,522
Total	255	253	51,666,020

*Source*: National Election Commission of South Korea (http://info.nec.go.kr)

*Note*: The entries are based on April 15, 2020, the date of the 21st National Assembly election.

That being said, electoral districts can be categorized into three types: “single-a,” “single-b,” and “multi.” In “single-a” cases, a legislator represents only one administrative unit, while in “single-b” cases, a legislator represents multiple administrative units. The “multi” type indicates cases where multiple legislators represent a single administrative unit.

For instance, administrative units like Jongro-gu in Seoul fall under the single-a type, where one legislator perfectly matches one administrative unit. In contrast, cases like “Sockcho-si-Inje-gun-Goseong-gun-Yangyang-gun” in Gangwon belong to the single-b category, where one legislator represents four administrative units (Sock-cho, Inje, Goseong, and Yangyang). Both single types involve one legislator representing one or two administrative units. On the other hand, Suwon-si in Gyeonggi is divided into five electoral districts: Suwon-si A, Suwon-si B, Suwon-si C, Suwon-si D, and Suwon-si E, where five legislators collectively represent one administrative unit. This multi-type district, exemplified by Suwon-si, is crucial for our hypotheses, as such electoral environments could potentially lead legislators to be less attentive to local spending due to the blurred clarity of responsibility toward constituents.

### 4.2. Data description

We acquired a dataset of official accounting reports detailing legislators’ political spending through OhmyNews’ datahub (github.com/OhmyNews/KA-money). The dataset comprises 1,419,704 cases of political spending by legislators, spanning from 2012 to 2022, encompassing members of the 19th to the 21st National Assembly. It is important to note that in the current publicly available version of the dataset, data is only accessible starting in 2012, corresponding to the records of legislators from the 19th Korean National Assembly onwards. We extracted records pertaining to district members (SMDs) and excluded proportional representation members (PRs) from the dataset to focus our analysis.

Additionally, data related to legislators’ personal information, such as electoral district, age, seniority, and party affiliation, was obtained from the official Korean National Assembly information portal website (open.assembly.go.kr). We merged the dataset containing legislators’ information with the dataset of legislators’ political spending records by matching their unique identifiers. This process resulted in the creation of a panel dataset spanning from 2012 to 2022, encompassing legislators’ information and their corresponding spending records.

### 4.3. Measures

#### 4.3.1. The dependent variable: Constituency-oriented spending

The dependent variable used in the analysis is the ratio of legislators’ constituency-oriented spending to their total spending. Constituency-oriented spending was defined as the total yearly expenses allocated to address local issues and engage with constituents within the legal bounds of political funds during legislators’ terms. Given that the total political expenses allocated to lawmakers are strictly regulated by the NEC and the Political Funds Act, a higher percentage of constituency-oriented spending can be interpreted as indicative of a lawmaker’s level of attention to addressing district issues within the constraints of limited financial resources.

The original dataset of political spending is structured into 11 divisions and 36 subdivisions, covering a range of expenses such as public meetings, transportation, office operations, advertising, personnel, and research and development. To isolate constituency-oriented spending, expenses related to public meetings addressing local issues and transportation for commuting and visiting legislators’ districts were identified by filtering the relevant spending categories and conducting keyword searches for “local (*Ji-Yeok* in Korean).” Subsequently, to calculate the ratio of legislators’ constituency-oriented spending to their total spending, the former was divided by the latter, and the result was then multiplied by 100 to express it as a percentage. Table 2 shows that the ratio of constituent-related expenditures fluctuates over time.

**Table 2:** Descriptions of constituency-oriented spending: The dependent variable (%).

Year	N	Mean	S.D.	Min	Max
2012	246	0.286	0.940	0	8.458
2013	247	0.292	0.923	0	9.445
2014	251	0.431	2.506	0	38.198
2015	245	0.223	0.553	0	3.603
2016	349	0.112	0.345	0	4.409
2017	254	0.323	0.802	0	6.540
2018	262	0.269	0.722	0	6.704
2019	253	0.313	1.184	0	14.704
2020	385	0.143	0.456	0	4.174
2021	253	0.155	0.483	0	4.190
2022	260	0.185	0.529	0	4.181
Total	3,005	0.239	0.999	0	38.198

*Note*: The South Korean National Assembly has a legally mandated number of 253 seats for its single-member districts. Due to by-elections, replacements of members, and vacancies that occur during the term of office, the actual number of seats at the table differs from the legal number. In the analysis, legislators’ term duration was controlled.

#### 4.3.2. Independent variables and moderators

The metric for the number of legislators per administrative unit was calculated as the total count of legislators within an administrative unit. For instance, if a legislator solely represents a single administrative unit (single-a type), it is coded as 1. Likewise, if a legislator represents multiple administrative units alone (single-b type), it is also counted as 1. In the case where multiple legislators concurrently represent a single administrative unit (multi type), such as four legislators, the count of legislators is 4, reflecting their shared representation of the same administrative unit.

Partisan homogeneity refers to the degree of similarity in terms of party affiliations among the legislators within a specific administrative unit. The measurement of this variable relies on the Herfindahl-Hirschman Index, which is computed by squaring the proportion of each political party in the administrative unit and subsequently aggregating the resultant values. The formula for partisan homogeneity is as follows:

$$Partisan\;Homogeneity=p_{1}^{2}+p_{2}^{2}+\cdots +p_{n}^{2},$$

where: $p_{n}=the\;share\;percentage\;of\;party\;n\;in\;a\;given\;administrative\;unit$

For instance, if an administrative unit comprises three districts, each represented by legislators A (Party X), B (Party Y), and C (Party Z), the administrative unit’s partisan homogeneity would be 0.333, drawing from the calculation: (1/3)^2^ + (1/3)^2^ + (1/3)^2^. The index ranges from 0 to 1, where a value of 1 indicates that all legislators in an administrative unit share the same partisan affiliation.

Legislators’ seniority was quantified by the number of times they have been re-elected and was included in the analysis as a continuous variable. Moreover, interaction terms between the number of legislators per administrative unit and partisan homogeneity, as well as between the number of legislators per administrative unit and legislators’ seniority, were also included in the analysis.

#### 4.3.3. Covariates

Several control variables are incorporated into the analysis. First, in consideration of the influential role held by local government governors in distributing intergovernmental transfers from central governments to local administrations [21, 24], we introduce a binary variable denoting the party affiliation alignment between legislators and the governor (referred to as *Si-Jang*/*Do-Ji-Sa* in Korean). A value of 1 signifies alignment within the same party, while 0 indicates a mismatch in party affiliation.

Moreover, as some of the previous studies have shown the significant negative relationship between the winning margin and legislators’ constituency service [2, 25–27], electoral vulnerability is considered a covariate. Using the outcomes of the previous election, this variable is calculated as follows:

$$Electoral\;vulnerability=1-[\frac{\left(Winners’\;votes-Runneup’s\;votes\right)}{valid\;votes}]$$

Furthermore, legislators’ personal characteristics such as term duration, age (and age-squared), party affiliation, and regionalism are considered. While the legally mandated number of seats for single-member districts stands at 253, the actual count may vary due to factors such as by-elections, member replacements, and vacancies that occur during the term of office. Additionally, owing to election cycles, legislators’ terms commence on May 30th rather than January 1st of the election year. Consequently, legislators’ term duration was incorporated into the analysis as a continuous variable, indicating the total days served in office on a yearly basis.

Additionally, it is plausible that older legislators may possess stronger connections with local voters due to their accumulated experience compared to legislators in younger generations. To address this, age was included as a continuous variable in the analysis. Additionally, age-squared values were incorporated to consider the potential non-linear relationship between age effects and the dependent variable.

Legislators’ party affiliation was also considered in the analysis. Despite political parties frequently changing names, as of 2022, South Korea has two major lines: the Democratic Party (liberal) and the People Power Party (conservative). Predecessor parties corresponding to these two lines from 2012 to 2022 were treated as the same party. This resulted in two binary variables: one for the Democratic line (1 = Democratic line, 0 = others) and the other for the Conservative line (1 = Conservative line, 0 = others).

Traditionally, South Korea demonstrates an ideological and partisan cleavage along regional lines. *Youngnam*, encompassing Daegu, Gyeongbuk, Busan, Ulsan, and Gyeongnam, tends to support conservative parties, while *Honam*, including Gwangju, Jeonbuk, and Jeonnam, favors left-leaning parties aligned with the Democratic line [28]. Accordingly, due to traditionally strong levels of regionalism, partisan homogeneity is expected to be particularly strong in these regions. Therefore, two dummy variables, *Youngnam* and *Honam*, were included as controls in the analysis.

Table 3 offers descriptive statistics of the variables included in the analysis.

**Table 3:** Descriptive statistics.

Variable	N	Mean	SD	Min	Max
The ratio of constituency-oriented spending (%)	3,005	0.239	0.999	0	38.198
The number of legislators per administrative unit	2,872	2.005	1.028	1	5
Partisan homogeneity	2,767	0.877	0.210	0.333	1
Seniority	2,777	2.179	1.271	1	8
Co-partisan governor	2,770	0.571	0.495	0	1
Electoral vulnerability	2,774	0.838	0.140	0.234	1.000
Term duration	3,005	309.722	99.012	0	365
Age	3,005	57.512	6.790	32	78
Age^2^	3,005	3353.773	778.843	1,024	6,084
Democratic Line Party	2,777	0.488	0.500	0	1
Conservative Line Party	2,777	0.433	0.496	0	1
*Youngnam*	2,777	0.263	0.440	0	1
*Honam*	2,777	0.116	0.320	0	1
Year	3,005	2017.141	3.103	2,012	2,022

#### 4.3.4. Analytical strategy

Given our dataset’s panel structure spanning from 2012 to 2022, with legislators identified as the unit, we employed regression models incorporating two-way fixed effects at the level of legislators and years. Our final regression model follows the least squares dummy variable (LSDV) approach and is expressed as follows:


$$Y_{it}=\sum_{i=1}^{n}\alpha_{i}+\beta_{1}{Number}_{it}+\beta_{2}{Partisan\;homogeneity}_{it}+\beta_{3}{Seniority}_{it}+\beta_{4}{Number}_{it}{Partisan\;homogeneity}_{it}+\beta_{5}{Number}_{it}{Seniority}_{it\;}+\sum \gamma \;{Control}_{it}+{Legislator}_{i}+{Year}_{i}+\varepsilon_{it}$$

Here, $\sum_{i=1}^{n}\alpha_{i}$ indicates constant variables, and *β*_1_, *β*_2_, and *β*_3_ are regression coefficients indicating the effects of the number of legislators per an administrative unit, partisan homogeneity per an administrative unit, and legislators’ seniority, respectively. The coefficients *β*_4_ and *β*_5_ signify interaction effects between the main explanatory variables. ∑*γ* represents the coefficients of control variables, where *i* denotes a legislator, *t* signifies a given year, and *ε*_*it*_ denotes the error term. By employing LSDV models, we can effectively isolate the influence of the explanatory variables on legislators’ constituency-oriented spending, all the while controlling for unobserved individual legislator-specific and year-specific factors.

Our analysis followed this procedure: Given our primary interest in examining the dynamics between multiple legislators within administrative units, particularly regarding their party affiliations and seniority, we initially analyzed truncated data consisting exclusively of multi-type districts across Models 1 to 3. Model 1 served as the baseline regression model without interaction terms, while Model 2 introduced an interaction term between the number of legislators per administrative unit and the partisan homogeneity per administrative unit. Model 3, the full model, included an additional interaction term between the number of legislators per administrative unit and seniority in the analysis.

Moreover, in Models 4 to 6, we analyze the robustness of the hypothesized relationship between legislators per administrative unit, partisan homogeneity, and seniority within the broader context by utilizing the complete dataset, which includes all types of districts (single-a, single-b, and multi). The regression modeling process was the same as that of Models 1 to 3.

## 5. Results

Table 4 displays the results of the regression analysis. In Model 1, findings seem to be consistent with our Hypothesis 1: the effect of the number of legislators per administrative unit is statistically significant (b = -0.415, se = 0.142, p = 0.004). Within an administrative unit, an increase in the number of legislators negatively correlates with legislators’ constituency-oriented spending.

**Table 4:** Results from LSDV regression analysis.

	Multi type only			All district types		
	(1)	(2)	(3)	(4)	(5)	(6)
	Coef. (SE)	Coef. (SE)	Coef. (SE)	Coef. (SE)	Coef. (SE)	Coef. (SE)
(a) The number of legislators per administrative unit	-0.415**	-0.717**	-0.360	-0.162	-0.545**	-0.275
	(0.142)	(0.232)	(0.246)	(0.095)	(0.196)	(0.205)
(b) Partisan homogeneity	-0.191	-1.167	-1.790**	-0.236	-1.291*	-1.619**
	(0.230)	(0.633)	(0.647)	(0.180)	(0.506)	(0.510)
(c) Seniority	-0.436	-0.474	0.006	-0.384*	-0.411*	-0.136
	(0.290)	(0.291)	(0.312)	(0.195)	(0.195)	(0.205)
(a) × (b)		0.396	0.678**		0.445*	0.625**
		(0.239)	(0.247)		(0.200)	(0.203)
(a) × (c)			-0.216***			-0.141***
			(0.052)			(0.033)
Co-partisan governor	0.101	0.102	0.110	0.136*	0.136*	0.156*
	(0.078)	(0.078)	(0.078)	(0.061)	(0.060)	(0.060)
Electoral vulnerability	-1.328*	-1.307*	-1.291*	-0.586	-0.580	-0.597
	(0.547)	(0.547)	(0.544)	(0.322)	(0.322)	(0.321)
Term duration	-0.000	-0.000	-0.000	0.000	0.000	0.000
	(0.000)	(0.000)	(0.000)	(0.000)	(0.000)	(0.000)
Age	0.133	0.135	0.042	0.162	0.165	0.139
	(0.127)	(0.127)	(0.128)	(0.086)	(0.086)	(0.086)
Age^2^	-0.001	-0.001	0.000	-0.001	-0.001	-0.001
	(0.001)	(0.001)	(0.001)	(0.001)	(0.001)	(0.001)
The Democratic Line Party	-0.135	-0.134	-0.006	0.036	0.034	0.050
	(0.318)	(0.317)	(0.317)	(0.200)	(0.200)	(0.199)
The Conservative Line Party	0.059	0.107	0.245	0.143	0.147	0.159
	(0.323)	(0.324)	(0.324)	(0.179)	(0.179)	(0.179)
*Youngnam*	0.571	0.578	0.547	0.250	0.282	0.304
	(0.662)	(0.661)	(0.658)	(0.576)	(0.575)	(0.573)
*Honam*	-	-	-	-0.867	-0.863	-0.688
				(1.062)	(1.061)	(1.058)
Year dummies	Y	Y	Y	Y	Y	Y
Legislator dummies	Y	Y	Y	Y	Y	Y
Observations	1,854	1,854	1,854	2,759	2,759	2,759

*Note*: Results come from LSDV regression models; Standard errors are in parentheses

*** p<0.001

** p<0.01

* p<0.05 (two-tailed); In Models 1 to 3, the estimated coefficient for Honam was omitted from the models. This omission may be attributed to collinearity, possibly due to the strong homogeneity in this region, where values are nearly close to 1 (0.913) across districts.

Hypothetically speaking, if a legislator belongs to an administrative unit divided by multiple districts and the unit is further divided by one additional district, resulting in the addition of one legislator compared to other districts of counterparts, the ratio of legislators’ expenditure for addressing constituents’ needs and demands may decrease by 0.4 percentage points relative to their total yearly spending. In real-world scenarios, if a legislator changes her electoral district through elections, she may allocate more attention to her constituents in a district belonging to a less fragmented administrative unit (such as Seodaemun-gu divided by two districts) compared to a situation where she is elected in a district belonging to a more fragmented administrative unit (such as Suwon-si divided by five districts). This increased attention may stem from the clearer responsibility towards constituents in the less fragmented administrative unit. This finding seems to be consistent with previous literature on the size of electoral districts and legislators’ personal vote-seeking behavior (e.g., [8, 17]). Furthermore, the underlying rationale is comparable to the logic of the “law of 1/n” in the literature of public choice theory, which emphasizes that a smaller geographic area for representation is closely associated with a greater inclination of representatives to focus their efforts on meeting the demands of their constituents.

In Model 2, the interaction term between the number of legislators per administrative unit and partisan homogeneity did not reach statistical significance. However, in Model 3, with the inclusion of an additional interaction term involving the number of legislators per administrative unit and seniority, both interaction terms became statistically significant. This implies that the influence of partisan homogeneity may vary depending on legislators’ seniority, a factor not fully considered in Model 2. Seniority may act as a moderator, influencing the relationship between the number of legislators per administrative unit and their efforts toward constituency services, as well as impacting how partisan homogeneity affects the distribution of legislators per administrative unit.

The findings of Model 3 support Hypothesis 2, which posited that the negative relationship between the number of legislators within an administrative unit and legislators’ personal vote-seeking efforts in their district may be more pronounced when the administrative unit is represented by legislators from different parties compared to when it is represented by multiple legislators belonging to the same party. The positive coefficient of the interaction term between the number of legislators per administrative unit and partisan homogeneity suggests this inclination (b = 0.678, se = 0.247, p = 0.006).

A legislator in an administrative unit with multiple legislators is more likely to reduce spending on constituency-oriented expenditures. However, if the administrative unit consists of districts represented by her co-partisans, the extent of this reduction in spending may be mitigated compared to situations where she belongs to an administrative unit with districts represented by legislators from different parties. We interpret that these differences stem from the obscured responsibility for constituents among multiple legislators and multiple parties due to the increased number of actors. Just as a larger number of legislators per administrative unit can obscure their responsibilities for local constituents, reduced partisan homogeneity among legislators within the unit may decrease their focus on constituency-related matters. This can occur because the environment makes it challenging for voters to identify which party contributes to local progress, consequently weakening legislators’ motivation for credit claims.

In Model 3, the negative coefficient of the interaction term between the number of legislators per administrative unit and legislators’ seniority also supports the validity of Hypothesis 3 (b = -0.216, se = 0.052, p = 0.000). This suggests that the negative relationship between the number of legislators per administrative unit and legislators’ personal vote-seeking efforts in their district may be more pronounced among legislators with greater seniority compared to newly-elected legislators. In other words, among newly-elected legislators, regardless of the number of other legislators in their vicinity, they tend to consistently maintain constituent-oriented spending.

**Fig 1:**
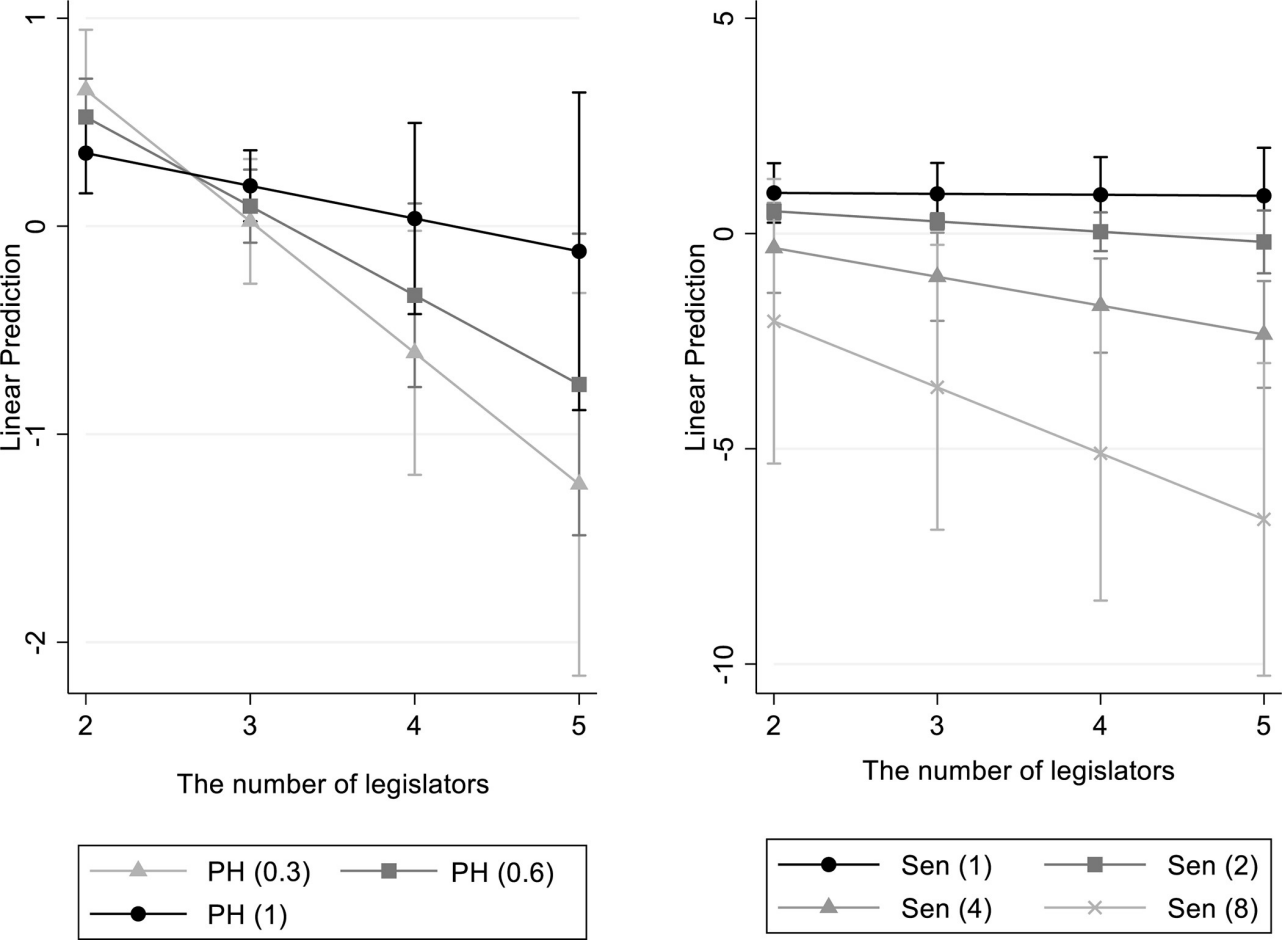
The number of legislators per administrative unit and legislators’ constituency-oriented spending. Note: The graphs presented above depict the outcomes of Model 3. In these graphs, the abbreviation “PH” represents partisan homogeneity per administrative unit, while “Sen” denotes legislators’ seniority.

Fig 1 graphically demonstrates the moderating effects of partisan homogeneity and legislators’ seniority on the relationship between the number of legislators per administrative unit and legislators’ constituency-oriented efforts based on the results presented in Table 4.

The outcomes of Models 4 to 6 were derived from the dataset encompassing all cases of legislators who represent not only multi-type districts but also single-a or single-b districts. It is noteworthy that in cases of both single-type districts, partisan homogeneity is 1, as well as the number of legislators per administrative unit being 1. While the results and interpretation from Models 1 to 3 are only applicable for examining cases of districts belonging to multi-type districts, Models 4 to 6 have the advantage of investigating whether the hypothesized relationship remains valid after comparing multi-type districts with single-type districts.

In Model 4, the estimated coefficient of the number of legislators per administrative unit was not significant. However, in Models 5 and 6, we found significant interaction terms indicating the moderating effects of partisan homogeneity and seniority, respectively. These findings indicate that Hypotheses 2 and 3 are supported when analyzing the complete dataset of all types of districts.

## 6. Concluding remarks

Using public records of Korean National Assembly members (2012−2022), the present study demonstrates that legislators are more likely to spend money on constituent services when the administrative unit to which their district belongs is represented by fewer legislators. This is presumably because in administrative units with fewer legislators, the clarity of each legislator’s obligation to constituents becomes more evident, thereby amplifying their motivation to maintain personal reputations and secure re-election. This finding aligns with the fundamental logic observed in existing literature, indicating that an increase in the number of actors corresponds to a decrease in their individual efforts [8, 17]. Furthermore, it resonates with the perspective of public choice theory, which underscores legislators’ motivation to appeal to their constituents, a tendency notably pronounced in smaller district sizes [10, 18].

Furthermore, this study underscores that the inverse relationship between the number of legislators within a district and their personal vote-seeking efforts may not uniformly apply. Rather, it posits variations based on partisan homogeneity and legislators’ seniority. Specifically, findings indicate that this negative correlation was more pronounced in districts represented by legislators from different parties compared to those from the same party. Additionally, it was more prominent among experienced legislators compared to newly-elected ones.

In spite of a meaningful contribution to the literature on legislators’ individual vote-seeking behavior, this study has a few weaknesses. First, though the analysis considers fixed-effects at the level of individual legislators and time, the findings are correlational, not causal. It hinders us from offering policy suggestions. If the main finding is causal and therefore correctly represents reality, then it can be incorporated into the process of redistricting and electoral reform. For example, when politicians (and voters alike) agree that a larger amount of pork provisions is necessary in order to increase voters’ well-being, districts should be drawn by being overlapped with other administrative units. Carefully designed experiments that manipulate partisan homogeneity need to be followed in order to yield relevant policy implications.

Second, albeit not causal, mechanisms between partisan homogeneity and legislators’ vote-seeking behavior have to be elaborated. The present study expects that when a legislator shares party affiliations with other elected officials in an administrative unit, she is more likely to collaborate with her colleagues. We assumed that this collaboration was due to a mutual interest among legislators of the same party to bolster their party’s reputation, particularly due to the heightened clarity surrounding party affiliation. However, it is also plausible to consider inverse reasoning. For instance, there is another potential scenario where a legislator may prefer not to collaborate but rather to compete with her co-partisan. This could be driven by a desire to distinguish herself from other members of the same party in order to maintain a higher individual reputation. Despite this possibility, the detailed reasons behind legislators’ behavior in this regard are not thoroughly examined in this study. Additional information from a survey or an interview of legislators and candidates is required to peek into the mechanism between partisan homogeneity and legislators’ vote-seeking behavior.

One of the legislators’ duties is to serve voters in their district. A way in which legislators cater to the interests of their constituency is to spend money. While the well-reported determinants of a legislator’s vote-seeking (and vote-securing) behavior include district size, there has been relatively little focus on the importance of administrative units as well as the partisan homogeneity within these units. Given that intergovernmental transfers from central governments to local administrations are distributed based on administrative units rather than electoral districts, this study proposes a shift in focus from electoral districts to administrative units. This transition enables the integration of the theory of legislators’ personal vote-seeking behavior with insights from public choice literature concerning the common pool problem. Moreover, the finding that partisan homogeneity and the political career trajectory of legislators could moderate the correlation between the number of legislators per administrative unit and their constituency-oriented spending will enrich discussions on electoral system reform, redistricting, and the nomination process within political parties.
